# Anatomy and Physiology of the Teeth

**Published:** 1869-08

**Authors:** James B. Hodgkin


### ARTICLE II.

### *Anatomy and Physiology of the Teeth.*

By James B. Hodgkin, D.D.S.

The teeth—their beauty as ornaments—their usefulness as organs—their characteristics; would make up a volume, much less an article of a few pages. Their Anatomy and Physiology is the theme of the present paper.

It is not necessary to give the divisions of incisors, &c., and passing over these and other preliminaries, we will briefly notice their physiological development, as seen in intra-uterine life, and trace this development into the tissues which compose them when erupted.

In the study of the anatomy (comparative) of the teeth, it would be interesting to note among the various classes of mammalia the differences in their structure and the various combinations of the elements (not ultimate elements, but those of structure, enamel, dentine &c.) which go to form that structure. It is impossible within the limits laid down for this paper to go into this investigation to any great extent, and I will content myself by merely stating that of the elements of tooth structure, dentine is the most common and is universally present in the body of the tooth. Enamel is much less constantly found,—is in fact often wanting entirely, and certainly where present is often dispensed with without injury. Cementum is generally found with dentine, and seems to stand as a sort of intermediate link between the hard, nearly inorganic dentine, and the subjacent soft tissues—a sort of compromise between bone and tooth.

In the 6th or 7th week of intra-uterine life, a ridge appears in the mouth of the fœtus where the coming maxilla is to be, the groove anterior to this ridge separating it from the lip, and posteriorly from the tongue &c.

Shortly this ridge is itself grooved, and in this (primitive dental) groove appear small papillæ, the germs of the deciduous teeth. These are soon partitioned off, one from the other, by septa which enclose them on the sides; the sides of the groove already enclosing them are the labial and lingual surfaces or rather sides. These are next covered in by opercula shooting over and meeting above them.

A curious provision is here made for the development of the permanent tooth. The operculum which advances toward the centre from the anterior portion of the maxilla closes in and fills up the space between the ridge and the papillæ; not so, however with the other, the posterior, for it, as it were, divides and leaves between its folds a cavity—the "cavity of reserve," in which is developed the permanent tooth, now in the rudimentary stage. These cavities with their contents, which are at first found behind and partly above the primitive or deciduous papillæ, by degrees descend until from having been as just described, *above* they pass *behind* and *below* the first, and stand ready to take their places in time. They are to be the permanent teeth, though seldom in this day retaining their claim to that designation throughout life.

Physiologically, the "six year," as they are termed, or the first permanent molars belong to the second dentition; but inasmuch as they arise from and are developed in the primitive dental groove they are anatomically assigned to the class of deciduous teeth; as they are not developed from "cavities of reserve," as are all other permanent teeth. In passing, it may be remarked that the second molars are developed from cavities of reserve cut off from the first permanent molars, and that the third (dens sapientiæ) are in turn developed from a cavity of reserve cut off from the second molar.

Let us for a few moments peer with curious eyes into the mysterious development going on within the dental capsule, which we have thus seen enclosed about the rudimentary tooth, and lay bare and understand, as well as we may, the hidden processes, and endeavor to comprehend its evolutions.

On the surface of the papilla is developed germinal matter, subsequently to be converted into dentine, and on the internal surface of the operculum germinal matter, to be developed into enamel is being also formed. The "germinal matter" of the dentine growing out from the papilla, meets that growing from the enamel organ, the point of contact being that of the first production of "formed material"—that is, the oldest formed material is on this line of meeting. It is at this point that calcification begins, and it proceeds *out* for the enamel, *in* for the dentine.

While the enamel is fully calcified, and rendered a mass practically inorganic and lifeless by that process, the same is not true of the dentine. It is not fully calcified, that is all the fibrillæ of "germinal matter" which form its structure do not harden, but many are left as soft intertubular masses, which seem organically continuous with the pulp. It is these that are sensitive in excavating dentine. Whether they are simply masses of "germinal matter" with endowments of pain conduction, or whether they are connected with the ultimate fibrillæ of nerves is a question over which much obscurity hangs. It is highly probable, however, that the nerve tissue of the pulp is organically continuous with these fibrillæ; at least there is no evidence that they do not thus terminate, and the most that can be said is that we have been unable to trace this connection. Much light needs to be shed on this subject before it is fully understood.

The fact that dentinal fibrillæ heretofore uncalcified may do so at any time is a curious and important one. In anatomical structure they are larger on the surface of the pulp than elsewhere, and as they radiate outward toward the periphery of the dentine they branch, subdivide, and anastomose until they are lost in infinitely small and imperceptible termini. I have said that they are subject to calcification, and this may go on (it probably never does, but there is no reason why it might not occur) until not only are the intertubular substances calcified, but the pulp itself, with which, as has been before stated, they seem organically continuous.

The "zone of consolidation" in caries is considered as a proof of the inherent power of the pulp to calcify, manifested under exciting causes. This may perhaps be only partly true, for we do not *know* that the pulp substance itself does calcify—we do not know but that a compound process goes on in this case, the sequel of which is the formation of calcified substance from germinal matter. It is true that practically it is not a matter of much importance as the pathological treatment would of necessity be unchanged.

Enamel in its development assumes the form of primitive hexagonal rods. It, different from dentine, calcifies as before stated throughout its substance. It has no intertubular substances, no power of repair—it is dead.

The cementum investing the root of the tooth, much resembles bone in its anatomical structure, with lacunæ, canaliculi, &c., but is more dense, and has fewer of those characteristics which so markedly distinguish bone. In cases where it becomes very dense, there seems to be an incompatability established between it and the surrounding soft tissues, which sometimes eventuates in loss of the organ. It is also the seat of the disease known as exostosis of roots, and sometimes when exposed is quite sensitive. It is invested with periosteum, and is nourished from it by germinal matter developed on the internal surface of that membrane, which is essentially a bone-feeder and producer.

The Pulp, consisting of arteries, veins, nerves, &c., held together by connective tissues, is a highly vascular structure, capable of high inflammation, and of exquisite sensibility. It is the organ of life to the tooth, its vital developing principle. To what extent the parts of the tooth adjacent to it (the pulp) are nourished by it, it is difficult to say. We know that its loss, death and removal, does not of necessity involve the loss of the tooth, and this has led some to the conclusion that it is only of use as an organ of development, its work being finished with that development complete.

But it is certain that nature never works thus clumsily, leaving in the organism a thing whose uses have ceased. That it serves to convey nutrient material to the intertubular, non-calcified portions of the dentine is quite certain, and we know that all sensation in the dentine ceases on its (the pulp's) destruction. We find also calcification going on slowly through the years of childhood, and of adult life to old age, the foramina of the nerve canals (pulp canals), which in early life are larger, growing smaller, and the pulp cavity itself decreasing in size; and in old subjects septa are found forming in the pulp canals in some cases, dividing them into secondary canals, or nearly obliterating their cavities. The "zone of consolidation" in caries would seem to imply either that a reserved power resides in the pulp for special contingencies, or that a special function is developed for an emergency.

The diseases of the Pulp belong to the domain of Pathology.

